# 504 The Geography of Community Integration for Burn Injury Survivors: A Comparative Analysis Across Multiple Metropolises

**DOI:** 10.1093/jbcr/iraf019.133

**Published:** 2025-04-01

**Authors:** Pyung Kim, Dohyeong Kim, Karen Kowalske

**Affiliations:** The University of Texas at Dallas; The University of Texas at Dallas; University of Texas Southwestern

## Abstract

**Introduction:**

This study investigates geospatial factors associated with community integration through the following research objectives: (1) explore geographic variations in community integration among burn injury survivors in four metropolises with Burn Model System (BMS) centers, (2) identify community-level social and environmental factors that influence integration, and (3) compare how these factors differ across the four cities.

**Methods:**

The primary data source for this study is the BMS National Database. Inclusion criteria were limited to adult burn survivors with injuries sustained between 2015 and 2022 and follow-up data at 6 months post-injury to ensure ZIP code availability for spatial analysis. This resulted in a final sample of approximately 1,500 adult burn survivors. The supplementary geospatial data was obtained from the Urban Institute’s Upward Mobility Data, which offers county-level metrics to assess community conditions over time using 19 variables across four categories: (1) Employment Opportunities, (2) Inclusive Neighborhoods, (3) Healthy Environment, and (4) Responsive and Just Governance.

Using this dataset, we performed four analyses: (1) Created county-level maps to illustrate average Community Integration Questionnaire (CIQ) scores before the injury and at 6 months post-injury; (2) Conducted Ordinary Least Squares (OLS) regression to determine which community characteristics were associated with reduced community reintegration outcomes for burn injury survivors; and (3) Examined how these factors varied across the four major metropolitan areas.

**Results:**

First, counties showing significant decreases in CIQ scores are predominantly rural. Second, these counties tend to exhibit unfavorable conditions, including low economic inclusion, weak social capital, poor transportation, and healthcare access, as well as lower environmental quality, neonatal health, and safety from trauma. Third, however, the strength of these associations with community-level characteristics varies significantly across the different metropolitan areas.

**Conclusions:**

First, the findings suggest that community integration is shaped not only by individual demographic attributes or health status but also by broader community-level factors. Second, the determinants of community integration vary significantly across regions, indicating the need for future research to explore the underlying mechanisms contributing to these regional disparities.

**Applicability of Research to Practice:**

This research provides actionable insights for physicians and healthcare practitioners involved in burn treatment and recovery. For example, the study highlights the importance of access to rehabilitation services, community support systems, and access to healthy foods in improving patient outcomes. These insights can inform clinical decision-making, guide treatment protocols, and enhance multidisciplinary approaches to patient care.

**Funding for the Study:**

This research was supported by Foundation Funding. The views expressed are solely those of the author and do not represent the views of the funding organization.

